# Silencing “Top-Down” Cortical Signals Affects Spike-Responses of Neurons in Cat’s “Intermediate” Visual Cortex

**DOI:** 10.3389/fncir.2017.00027

**Published:** 2017-04-25

**Authors:** Jin Y. Huang, Chun Wang, Bogdan Dreher

**Affiliations:** ^1^Discipline of Anatomy and Histology, The University of SydneySydney, NSW, Australia; ^2^Discipline of Biomedical Science, School of Medical Sciences, The University of SydneySydney, NSW, Australia; ^3^The Bosch Institute, The University of SydneySydney, NSW, Australia

**Keywords:** peristriate cortex, area V3, infero-temporal cortex, reversible inactivation, feedback from higher-order cortices

## Abstract

We examined the effects of reversible inactivation of a higher-order, pattern/form-processing, postero-temporal visual (PTV) cortex on the background activities and spike-responses of single neurons in the ipsilateral cytoarchitectonic area 19 (putative area V3) of anesthetized domestic cats. Very occasionally (2/28), silencing recurrent “feedback” signals from PTV, resulted in significant and reversible reduction in background activity of area 19 neurons. By contrast, in large proportions of area 19 neurons, PTV inactivation resulted in: (i) significant reversible changes in the peak magnitude of their responses to visual stimuli (35.5%; 10/28); (ii) substantial reversible changes in direction selectivity indices (DSIs; 43%; 12/28); and (iii) reversible, upward shifts in preferred stimulus velocities (37%; 7/19). Substantial (≥20°) shifts in preferred orientation and/or substantial (≥20°) changes in width of orientation-tuning curves of area 19 neurons were however less common (26.5%; 4/15). In a series of experiments conducted earlier, inactivation of PTV also induced upward shifts in the preferred velocities of the ipsilateral cytoarchitectonic area 17 (V1) neurons responding optimally at low velocities. These upward shifts in preferred velocities of areas 19 and 17 neurons were often accompanied by substantial increases in DSIs. Thus, in both the primary visual cortex and the “intermediate” visual cortex (area 19), feedback from PTV plays a modulatory role in relation to stimulus velocity preferences and/or direction selectivity, that is, the properties which are usually believed to be determined by the inputs from the dorsal thalamus and/or feedforward inputs from the primary visual cortices. The apparent specialization of area 19 for processing information about stationary/slowly moving visual stimuli is at least partially determined, by the feedback from the higher-order pattern-processing visual area. Overall, the recurrent signals from the higher-order, pattern/form-processing visual cortex appear to play an important role in determining the magnitude of spike-responses and some “motion-related” receptive field properties of a substantial proportion of neurons in the intermediate form-processing visual area—area 19.

## Introduction

**Figure 1:**
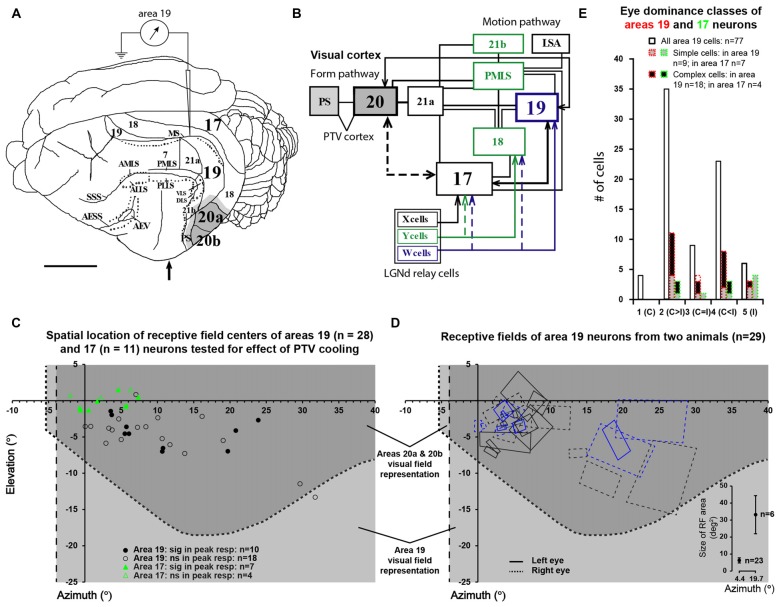
**(A)** The dorsolateral view of cat’s left cerebral hemisphere with approximate location of electrode penetrations in area 19. Upward arrow indicates approximate position of the Horsley-Clarke anterior-posterior zero coordinates. Dark gray area indicates the location of the foot of the cooling probe which covered most of areas 20a and 20b. Lighter gray indicates the spread of cooling in the vicinity of cooling foot. **(B)** A block diagram of the cat’s visual system with simplified neuronal circuitry of retino-geniculo-cortical pathways, pattern/form and motion processing cortico-cortical hierarchies. Note that many cortical areas including area 19 and postero-temporal visual (PTV) cortex are directly interconnected via horizontal cortico-cortical feedforward and feedback connections (after Rosenquist, 1985; Dreher, 1986; Salin and Bullier, 1995; Scannell et al., 1995; Dreher et al., 1996). **(C)** The location of receptive field (RF) centers of 28 area 19 neurons tested for the effects of cooling of PTV. Note the location of RF centers of 11 area 17 neurons examined for velocity-tuning in the previous series of experiments (Huang et al., 2007). **(D)** Outlines of RFs of area 19 neurons of two cats included in the present study. The effects of cooling PTV were tested only in some cells. The bottom right insert plots area 19 cells’ average RF areas at azimuths around 5° and 20°. **(E)** Frequency distribution of eye dominance classes of area 19 cells recorded in the present study (solid lines) and the cooling sample (red dotted lines). Area 17 data are shown in green.

The location and pattern of interconnections between neocortical areas processing visual information in the eutherian carnivore, domestic cat, are illustrated in Figures 1A,B. As in other mammals studied so far: (1) “lower-order” visuotopically organized cortical areas, some of which receive their principal or a substantial, direct thalamic input from the dorsal lateral geniculate nuclei (LGNd), send numerous “feedforward” associational projections to the “higher-order” visual areas; (2) beyond the primary visual cortices, information about pattern/form vs. motion is processed along two largely parallel “quasi-hierarchical” feedforward streams; and (3) higher-order areas send numerous associational “recurrent” or “feedback” projections back to lower-order areas (cat: Rosenquist, 1985; Dreher, 1986; Salin and Bullier, 1995; Scannell et al., 1995; Dreher et al., 1996; macaque monkey: Van Essen and Maunsell, 1983; Felleman and Van Essen, 1991; Bullier, 2004; Nassi and Callaway, 2009; Gilbert and Li, 2013; Markov and Kennedy, 2013; rat: Sanderson et al., 1991; Coogan and Burkhalter, 1993; Johnson and Burkhalter, 1996; mouse: Wang and Burkhalter, 2007; Wang et al., 2012).

The direct connections between visual areas are reciprocal but not strictly hierarchically sequential. In particular: (1) primary visual cortices (striate cortices, areas 17, areas V1) at the very bottom of the postulated hierarchies, send direct feedforward projections not only to areas at the immediate next level of putative hierarchies but also to areas higher-up; and (2) areas at the intermediate levels of the putative hierarchy send their direct feedforward and feedback projections not only to their immediate hierarchical neighbors but also to hierarchically more remote areas, including the primary visual cortices (Dreher, 1986; Felleman and Van Essen, 1991; Markov and Kennedy, 2013; Markov et al., 2014).

The cat’s postero-temporal ventral (PTV) cortex encompasses cytoarchitectonic areas 20a and 20b (Tusa and Palmer, 1980). PTV constitutes a distinct part of pattern/form information processing stream (Campbell, 1978; Lomber et al., 1996a, b) and is the presumed homolog of primate infero-temporal cortex (Payne, 1993). Although PTV and primary visual cortices are interconnected only indirectly (Figure 1B, Rosenquist, 1985; Dreher, 1986; Dreher et al., 1996; Burke et al., 1998; Batardiere et al., 1998), reversible inactivation of PTV affects the responses and a number of receptive field (RF) characteristics, of a substantial proportion of neurons in the part of V1 corresponding visuotopically to PTV (Bardy et al., 2006, 2009; Huang et al., 2007).

Cytoarchitectonic area 19 (peristriate cortex, presumed homolog of V3 of primates—Payne, 1993; Rosa and Manger, 2005) and cytoarchitectonic area 21a (presumed homolog of V4 of primates—Payne, 1993), like PTV, constitute distinct parts of the pattern/form information processing cortical stream (area 19: Sprague et al., 1977; Khayat et al., 2000; reviews Orban, 1984; Dreher, 1986; Dreher et al., 1996; area 21a: Dreher, 1986; Mizobe et al., 1988; Wimborne and Henry, 1992; Dreher et al., 1993; Lomber et al., 2001; Villeneuve et al., 2009). Both areas 19 and 21a have substantial direct interconnections with the primary visual and PTV cortices, and are likely therefore to play important roles in shaping the exchange of information between PTV and V1 (Figure 1B, for reviews, see Rosenquist, 1985; Dreher, 1986; Dreher et al., 1996; Batardiere et al., 1998). Indeed, reversible inactivation of entire area 21a and part of area 19, results in substantial changes in the magnitude of responses and many RF properties of large subsets of neurons in the visuotopically corresponding part of V1 (Wang et al., 2000).

A substantial amount of data has been accumulated concerning the role of feedback from the higher-order visual areas on the responses of V1 neurons (for review see Gilbert and Li, 2013). However, very little is known about the role of feedback from the higher-order visual areas on the responses of neurons in the intermediate areas and putative role played by those areas in shaping the exchange of information between the higher-order areas and V1. Thus, in the present study, we examined the effects of reversible inactivation by transient cooling to 10°C of ipsilateral PTV cortex, on the responses of neurons located in the intermediate part of pattern/form information processing stream—area 19. In a substantial proportion of these cells, PTV inactivation affected the magnitude of responses to optimal and peri-optimal visual stimuli and/or induced upward changes in velocity preferences and direction selectivity indices (DSIs), that is, the properties which are thought to be determined by the properties of their dorsal thalamic and/or feedforward cortical inputs.

## Materials and Methods

Animal preparation and recording procedures followed the guidelines of the Australian Code of Practice for the Care and Use of Animals for Scientific Purposes and were approved by the Animal Care Ethics Committee of The University of Sydney. The effects of PTV cooling on background activity and responses of area 19 cells were tested in four adult female cats (*Felis catus***)** weighing 2.5–3.4 kg. Animals were initially anesthetized with a gaseous mixture of 2.5%–5% of halothane in N_2_O/O_2_ (67%/33%). Throughout subsequent surgery, halothane level in the mixture was reduced to 1%–2%, while throughout the single-neuron recording sessions and between the intervals, halothane level was kept within 0.4%–0.8%. A tracheotomy was performed in order to reliably maintain artificial ventilation. Right cephalic vein was cannulated to allow continuous slow infusion of nutrients and paralyzing agent gallamine triethiodide injected at a rate of 10 mg/kg/h. Gallamine triethiodide-block resistant, residual eye movements were largely eliminated by bilateral cervical sympathectomy (see Rodieck et al., 1967). Opportunistic infections, brain swelling and excessive mucous secretion were controlled by daily injections of respectively the antibiotic amoxycillin trihydrate (75 mg, i.m.), dexamethasone phosphate (4 mg, i.m.) and atropine sulfate (0.3 mg, i.m.). Body temperature (37.5°C), heart rate (180–220 beats/min), alveolar CO_2_ level (3.5%–4%) and electroencephalogram (δ waves 0.5–4 Hz) were continuously monitored and maintained at appropriate levels. Phenylephrine hydrochloride (0.1%) was applied to each eye to block accommodation and to retract nictitating membranes. Pupils were dilated by daily applications of a couple of drops of atropine sulfate (1%). Neutral-power, air-permeable plastic contact lenses (radius 8.5 mm) were fitted to protect the corneas from drying. Artificial pupils (3 mm in diameter) and corrective lenses were placed in front of the eyes. Refractive power of corrective lenses necessary to focus the eyes on a tangent screen located 57 cm in front of the cat was determined by slit/streak retinoscopy (range: +0.5 to +3.0 diopters). In order to monitor the residual eye movements, positions of optic discs and *areae centrales* (Bishop et al., 1962) were, back-projected, at least twice daily, onto the tangent screen using a fiber-optic light source (Pettigrew et al., 1979).

Following an injection of 10 ml of D-mannitol (25%; i.v.), separate craniotomies were performed over the left ventral posterior suprasylvian region (Horsley**-**Clarke or HC coordinates: P3–P8 mm, lateral 15–20 mm) and left area 19 (HC coordinates: A2–P8 mm, lateral 2–12 mm). Between cooling sessions, silver foot temperature was maintained at 36°C.

A craniotomy in stereotaxic coordinates was positioned according to the visuotopic map of area 19 (Tusa et al., 1979). A plastic cylinder around it was glued with dental acrylic forming a well. Then a small opening was made in the dura mater where stainless steel microelectrodes (impedance 7–12 MΩ; FHC, Bowdoinham, ME, USA) were positioned vertically above the recording site. The well was filled with 4% agar in physiological saline then sealed with warm liquid wax (melting point **~**40°C). Using a hydraulic micromanipulator (David Kopf’s Instruments Tujunga, CA, USA) the electrode was advanced slowly (20–60 μm/min) along the medial suprasylvian sulcus (MS in Figure 1A).

A Peltier device attached to a specially designed silver probe was used to reversibly inactivate areas 20a and 20b (Tusa and Palmer, 1980) by cooling the probe to ~10°C, and subsequently rewarming it back to 36°C (Figure 1A). The foot of the probe covered almost all of areas 20a and 20b located on the dorso-lateral surface of cerebral hemisphere and the part of posterior suprasylvian area behind posterior suprasylvian gyrus (Figure 1A). The probe and the opening were sealed with vacuum grease, which also acted as a thermal insulator. At the end of each experiment, cooling probe’s location was checked by careful inspection of indentation marks left by the probe on the dura over PTV. We related these marks to the location of areas 20a and 20b (Tusa and Palmer, 1980) and PS area (Updyke, 1986). In several control experiments, we monitored the temperature and neuronal activity of the cortex under and around the cooling probe using a pair of micro-thermo-couples (25 μm thick wires) glued to the recording electrode. Monitoring was conducted before, during and after rewarming PTV cortex at various (0–11 min) time intervals and at various depths below the cortical surface.

The computer-controlled screen monitor located 57 cm in front of the cat’s eyes was usually set at a luminance of 15 cd/m^2^ against a background of 1 cd/m^2^. The stimuli—elongated light bars were set at cell’s RF center. The extracellular action potentials from single neurons were recorded, conventionally amplified and monitored both visually and acoustically (via a loud speaker). To exclude multiunit data, special attention was paid to the shape and magnitude of the action potentials. Only identified single unit potentials triggered standard pulses which were then fed into a microcomputer for data collection.

After single-neuron data collection, the probe and underlying PTV were cooled (within 2 min) to 10°C and kept at this temperature throughout the period (15–18 min) of repeated data collection. Subsequently, the probe and underlying PTV was rapidly (~2 min) rewarmed back to 36°C. Then at set times (10, 30, 45 and if necessary 60 min) after rewarming PTV covering probe to 36°C, the same tests were repeated again.

Quantitative assessment of RF properties was based on the analysis of responses to stimuli presented via the dominant eye. To prevent cell habituation (or adaptation), a delay of 800 ms was introduced when the stimulus velocity exceeded 6.6°/s. In all runs in which the stimulus velocity did not exceed 33°/s, the bin width was 22 ms. In runs in which the stimulus velocity was higher, the bin width was 6 ms. Thus, the same number of spikes per bin at velocities ≥46.2°/s (bin width 6 ms) and at velocities ≤33°/s (bin width 22 ms) do not indicate the same or similar number of spikes/s. Optimally oriented visual stimuli were usually traveling at its preferred velocity across the RF in the direction orthogonal to its optimum orientation. Peri-stimulus time histograms (PSTHs) were constructed from a sum of 5 or 10 repeats for each test condition. A Gaussian weighted average over five neighboring bins of PSTHs were then applied to smooth the responses. Peak response rates and mean background activity were determined at each condition, tuning curves as well as widths at half-height (WHH, see Figure 3Aii) were calculated.

**Figure 2:**
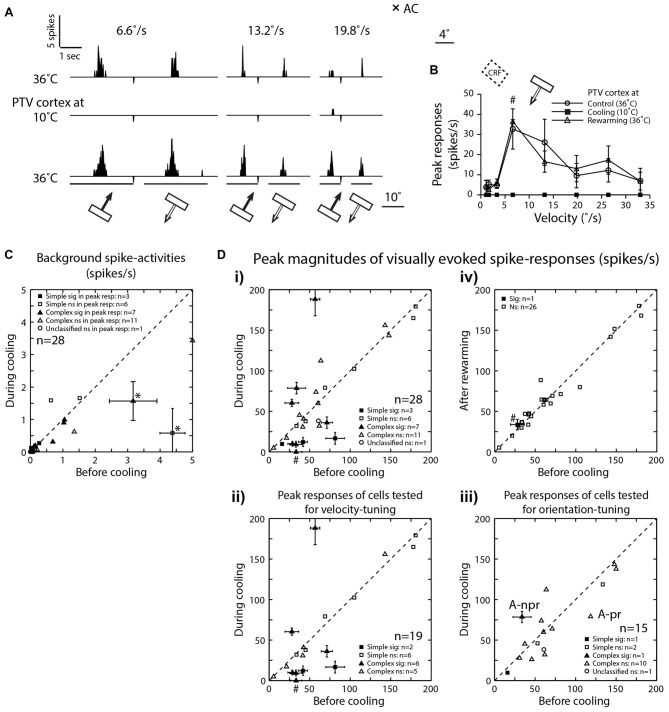
**(A)** Peri-stimulus time histograms (PSTHs) of spike-responses of a class 2 complex area 19 cell, to optimally oriented bar moving at different velocities across it’s RF. Upper row—before cooling, middle row—during cooling and lower row—after rewarming. Note, the absence of background spike-activity in all conditions. **(B)** Effects of cooling on peak magnitudes (spikes/s) of visually evoked responses of an area 19 neuron whose responses are illustrated in **(A)**. **(C)** Effects of cooling on visually evoked background spike-activities of area 19 neurons. Note that only in two cells (*) there was significant reduction. **(D)** The peak magnitudes of visually evoked responses before cooling PTV vs. those during cooling **(i)**. **(ii)** Cells tested for velocity-tuning. **(iii)** Cells tested for orientation-tuning. A-pr and A-npr refer to responses of cell in Figure 3A at preferred and non-preferred directions respectively. Note that the same cell is represented twice.** (iv)** Responses after rewarming PTV—all but one cell exhibited significant recovery. # In **(i, ii** and **iv)** denote responses indicated by # in **(B)**.

**Figure 3:**
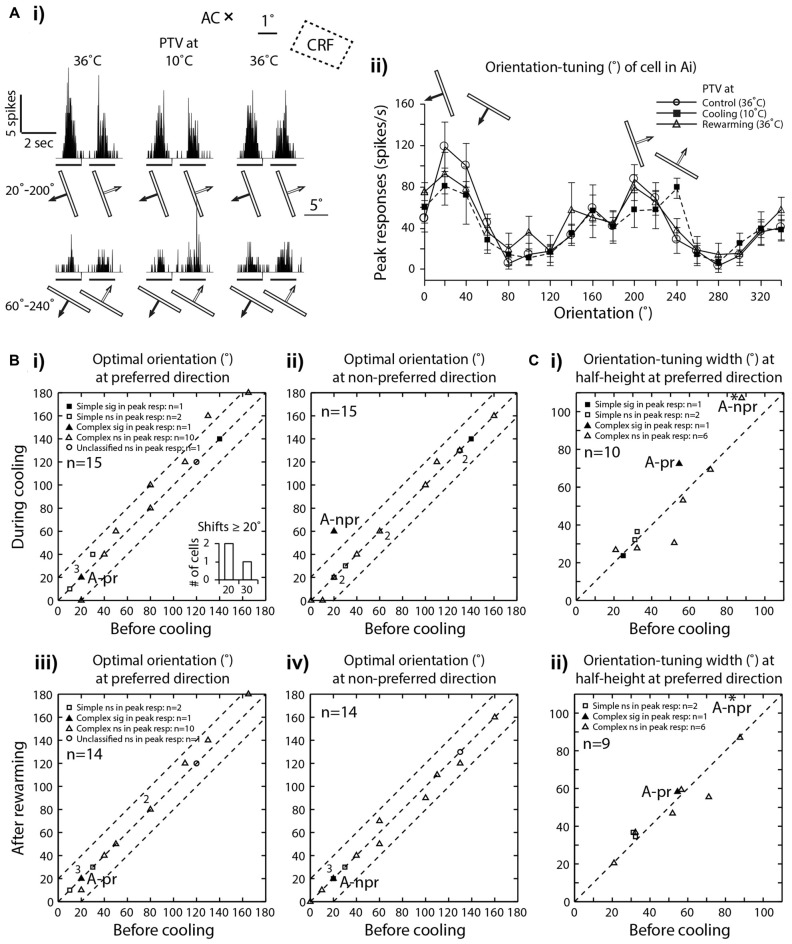
**(Ai)** PSTHs of spike-responses of an area 19 complex, class 2 cell, illustrating the influence of cooling of PTV on peak spike-responses to moving stimuli of two different orientations. Note that during PTV inactivation the cell exhibited substantial *decrease* in its peak responses to stimuli moving along the axis of one orientation (20°–200°) and a substantial *increase* in its peak responses to stimuli of another orientation (60°–240°). Stimulus size: 10° × 0.6°. **(Aii)** Effects of PTV cooling on orientation-tuning property of the same cell shown in **(Ai)**. Note during cooling, there was a 40° shift in optimal orientation but only in the non-preferred direction of movements. The shift was accompanied by an increase in orientation-tuning width at half-height (WHH). Thirty minutes after PTV was rewarmed, the responses and optimal orientation recovered to the pre-cooling levels. **(B)** Optimal orientations prior to cooling vs. those during cooling(**i**—preferred direction, **ii**—non-preferred direction) or after rewarming of PTV (**iii**—preferred direction, **iv**—non-preferred direction). In the insert frequency histogram within **(Bi)**, cells exhibiting large (≥20°) shifts in optimal orientation during inactivation of PTV are represented. Note that the numbers indicate the number of data points that overlap.** (C)** The widths of orientation-tuning curves at half-height calculated from the control condition vs. those during PTV cooling **(i)** and control condition vs. those after PTV rewarming **(ii)** * in **(Ci)** and **(Cii)** indicate a cell whose responses are illustrated in **(Ai)** and **(Aii)**. A-pr and A-npr refer to responses of cell in panel **(A)** at preferred and non-preferred directions respectively. Note the same cell is represented twice. The responses in non-preferred direction is indicated by * -npr.

The DSI was calculated according to: [(*R*_p_ − *R*_np_)/*R*_p_] × 100% where *R*_p_ and *R*_np_ are the peak discharge rates (in spikes/s) to optimally oriented stimuli moving in respectively preferred and anti-preferred directions along the axis perpendicular to the cell’s preferred orientation.

Statistical comparisons of the background spike-activity and magnitude of spike-responses between two sets of data were made using non-parametric tests. Using Mann-Whitney *U* test, for each cell, comparisons were made between successive control runs, control vs. PTV cooled runs, control vs. PTV rewarmed runs and PTV cooled vs. PTV rewarmed runs (Siegel, 1956). For comparisons of activities of groups of cells, we used Wilcoxon pair-matched signed-rank (referred to as Wilcoxon test; Siegel, 1956). In all comparisons a *P* value of the difference between two sets of data was ≤0.05 at the two-tailed criterion was considered to be significant. The ±values in the text are the standard deviations. In the Figures, bars indicate the standard error of the mean (SEM).

Experiments were terminated by deeply anesthetizing the animals with sodium pentobarbitone (Nembutal). Animals were perfused transcardially with Hartmann’s solution (1.2 L at 36°C), followed by 1 L of 4% paraformaldehyde in phosphate buffer (0.2 M at pH 7.4). In order to reconstruct the recording sites, brains were sectioned coronally at 50 μm, and stained with cresyl violet.

## Results

### Basic Characteristics of Area 19 Sample before Inactivation of PTV Cortex

The centers of the RFs (classical RFs − CRFs) of all 66 area, 19 cells studied were located within the contralateral visual hemifield in the region 0° − 32° from the vertical meridian and 2° − 15° from the horizontal meridian. The CRFs of all but two of 28 area 19 neurons examined for the effects of PTV inactivation were located within the part of the visual field represented in areas 20a and 20b (Figure 1C based on Tusa and Palmer, 1980). Our electrode penetrations proceeded along the middle suprasylvian sulcus and our sample is strongly biased toward particular cellular layers. Indeed, all but a couple of area 19 cells were recorded from the middle layers 3 and 4. Consistent with previous studies (see Tusa et al., 1979; Dreher et al., 1980; Duysens et al., 1982b; Rapaport et al., 1982; Dreher, 1986; Mulligan and Sherk, 1993), the RFs located more peripherally tended to be larger (Figure 1D). As indicated in Figure 1E, the majority of cells (85.5%; 66/77) were binocular and could be activated by appropriate stimuli presented via either eye (see Duysens et al., 1982a; Rapaport et al., 1982; Dreher, 1986; Pettigrew and Dreher, 1987; Tanaka et al., 1987; Guillemot et al., 1993; Tardif et al., 1997; Bergeron et al., 1998). About half of binocular cells (53%; 35/66) responded more strongly to stimuli presented via the contralateral eye (class 2 vs. class 4 cells—Figure 1E) with only a few cells (9/66; 13.5%) responding equally well to stimuli presented via either eye (class 3 cells). Note that the eye dominance distribution of area 19 cells tested for the effects of PTV inactivation was similar to that of the entire sample (Figure 1E).

The majority of neurons (64.3%; 18/28) examined for the effects of PTV cooling were classified as complex cells: they had spatially overlapping on and off discharge regions to optimally oriented stationary flashing light bars and/or spatially overlapping discharge regions to moving optimally oriented bars darker and brighter than the background. Simple cells, characterized by spatially non-overlapping on and off discharge regions, and/or spatially non-overlapping discharge regions to moving optimally oriented bars darker and brighter than the background, constituted about a third (32.1%; 9/28) of the sample. We were unable to classify one cell (1/28; 3.5%) on the basis of the above criteria.

It is worth noting that in the first study of RF properties of area 19 neurons of anesthetized cat (Hubel and Wiesel, 1965), no simple cells were reported (see also Guillemot et al., 1993). On the other hand, the proportions of simple or simple-like (see S cells, Sh cells and A cells of Henry, 1977) cells defined on the basis of existence of spatially distinct on (light bar) and/or off (dark bar) discharge regions are quite similar (25%—Dreher, 1986; Pettigrew and Dreher, 1987; to 31%—Duysens et al., 1982b) to that reported here. However, the reported proportions of simple-like cells defined on the basis of modulation ratio of the responses to moving luminance-modulated sinusoidal achromatic gratings (Skottun et al., 1991) with one exception (27%—Tanaka et al., 1987) were much lower (~10%—Tardif et al., 1997; Bergeron et al., 1998; Mimeault et al., 2002).

### Effects of Inactivation of PTV Cortex

#### Background Activity

In the majority of neurons (78.5%; 22/28), the background (“spontaneous”) activity was <1 spike/s (Figures 2A,C). Before PTV inactivation, the mean background activities of simple and complex cells were almost identical (mean: 0.78 spikes/s vs. 0.72 spikes/s; see Table 1). There was some reduction in the background activities of both simple (mean: 0.48 spikes/s) and complex (mean: 0.44 spikes/s) cells during PTV inactivation. However, only in a couple of cells (2/28; 7%—1 simple, 1 complex), the background activities during PTV inactivation were significantly lower than those before inactivation (Figure 2C). The mean background activities after rewarming PTV were lower but not significantly different from those before cooling (Table 1).

**Table 1:** **Background and peak-response spike-activities**.

	Condition	Mean ± SD (spikes/s)	Range (spikes/s)	Cell number	Statistical significance (Wilcoxon test)
**Background spike-activity**
All cells	Control	0.72 ± 1.32	0–5.01	*n* = 28	↓ *P* = 0.01*
	Cooling	0.46 ± 0.78	0–3.43
	Rewarming	0.49 ± 0.90	0–3.54	*n* = 27	↓ *P* = 0.08 (control vs. rewarming)
Simple cells	Control	0.78 ± 1.44	0.02–4.38	*n* = 9	↓ *P* = 0.92
	Cooling	0.48 ± 0.68	0–1.66
Complex cells	Control	0.74 ± 1.33	0.01–5.01	*n* = 18	↓ *P* = 0.005* (highly significant)
	Cooling	0.47 ± 0.86	0–3.43		
	Rewarming	0.57 ± 1.04	0–3.54		↓ *P* = 0.29 (control vs. rewarming)
**Peak-response spike-activity**				
All cells	Control	63.53 ± 46.52	5.42–180.75	*n* = 28	↓ *P* = 0.27
	Cooling	63.29 ± 57.03	0–188.30		
	Rewarming	65.84 ± 44.61	5.33–179.98	*n* = 27	↑ *P* = 0.41 (control vs. rewarming)
Simple cells	Control	83.39 ± 60.55	15.95–180.75	*n* = 9	↓ *P* = 0.055
	Cooling	70.51 ± 65.59	9.75–179.34		
	Rewarming	85.76 ± 56.41	36.92–179.98	*n* = 8	↑ *P* = 0.25 (control vs. rewarming)
Complex cells	Control	54.47 ± 38.46	5.42–147.83	*n* = 18	↑ *P* = 0.95
	Cooling	62.84 ± 56.60	0–188.30		
	Rewarming	57.06 ± 38.27	5.33–151.67		↑ *P* = 0.06 (control vs. rewarming)

#### Magnitude of Responses

The mean peak-firing rate of 28 area 19 neurons before PTV inactivation at 63.55 spikes/s was almost identical to that (63.3 spikes/s) during PTV cooling. However, in a quarter of cells (7/28; 4 complex; 3 simple), inactivation resulted in significant reduction (Figures 2Di, 3Ai,ii), or a complete cessation (Figures 2A,B) of responses to stimuli. In another quarter of the sample (7/28; Figure 2Di) the reductions in magnitude of responses were not significant. The last group included two cells (both complex—one binocular class 4, the other monocular class 5) whose RFs were located at the very periphery of the part of the contralateral hemifield represented in areas 20a and 20b (Figure 1C).

On the other hand, in almost a third of the sample (28.5%; 8/28 cells, all but one complex), inactivation of PTV resulted in *increases* in the magnitude of spike-responses to the optimized stimuli (Figures 2Di, 4A,B) and the increases were significant in ~10% (3/28, all complex) of the sample (Figure 2Di). In 2/19 cells in which effects of cooling on velocity preferences were tested, inactivation resulted in a clear enhancement of responses to stimuli moving at higher velocities (see Figure 2Dii, **Preferred velocities**). For a subset of cells tested for orientation-tuning, their peak responses are shown in Figure 2Diii. Note that only in one of those cells, there was a significant increase in peak firing rate during cooling (Figure 2Diii—**A-npr**).

**Figure 4:**
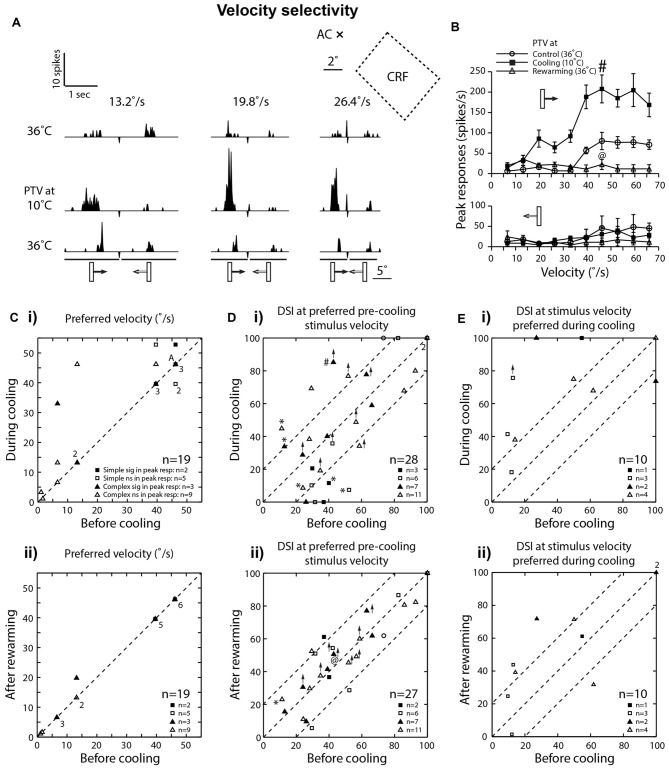
**(A)** Effects of PTV cooling on PSTHs of spike-responses of a class 2 complex area 19 cell to optimally oriented bar moving at different velocities. The stimulus was presented via the dominant (contralateral) eye. Stimulus size: 5° × 1°. **(B)** Velocity-tuning of peak spike-responses of the same cell whose responses are shown in **(A)**. Note in **(A,B)** there is a substantial *increase* in the magnitude of responses during inactivation of PTV, especially for movement from left to right. **(C)** Preferred velocities of area 19 cells before inactivation vs. those during PTV inactivation **(i)** and control vs. rewarming **(ii)**. In most cases, during cooling, inactivation resulted in upward shifts in preferred velocities. Note that the numbers indicate the number of data points that overlap.** (Di)** The direction selectivity indices (DSIs) of area 19 cells at stimulus velocities optimal before inactivation. In a large proportion of cells, inactivation resulted in large (≥20%) changes in their DSIs. Also in five cells (*) inactivation resulted in reversal of preferred directions of movements. # Denotes the response indicated by # in upper panel of **(B)**. **(Dii)** DSIs at stimulus velocities optimal after PTV rewarming. @ Denotes the response indicated by @ in upper panel of **(B)**. **(Ei)** The DSIs of a subpopulation of area 19 cells calculated at stimulus velocities optimal during cooling. Note in a majority of cells, inactivation resulted in large increases in their DSIs. **(Eii)** DSIs at stimulus velocities optimal during cooling in control condition vs. those after PTV rewarming.

In a substantial proportion of cases (6/15, 40%), cooling of PTV resulted in *decreases* in the magnitude of spike-responses to particular visual stimuli as well as *increases* in the magnitude of spike-responses to some other visual stimuli. Thus, in one cell, small significant *reductions* in the magnitude of spike-responses to optimally oriented moving light bar were accompanied by stimulus-direction dependant, significant *increases* in response to para-optimally oriented moving light bar (Figure 3Ai,ii; see effect on **Orientation-tuning**). Similarly, inactivation induced *reductions* in the magnitude of spike-responses to a light bar moving in one direction were accompanied by large and significant *increases* in the magnitude of spike-response to the same stimulus moving in the opposite direction (Figures 4A,B; see effect on **Direction selectivity**). The direction sensitive enhancement of response was even greater at higher velocities (Figures 4A,B; see effect on **Direction selectivity** and **Preferred velocities**).

With very few exceptions (Figures 2Div, 4A,B), rewarming resulted sooner or later (mean: 36 ± 20.07 min; range: 10–60 min) in virtually complete recovery of the responses to the control levels (Figures 2A,B,Div, 3Ai,ii).

### Receptive Field Properties

#### Silent Suppressive Regions

The RFs of a quarter (3/12—2 complex, 1 simple) of area 19 cells tested for length selectivity, contained clear-cut silent strongly suppressive regions along the axis of optimal orientation. In these termed end-stopped cells, stimulation of suppressive regions resulted in ≥50% reduction in the number of spikes (see hypercomplex cells of Hubel and Wiesel, 1965; see also Dreher, 1972). The proportion of end-stopped cells in our sample is at the lower end of the range reported previously (25%–38%: Hubel and Wiesel, 1965; Duysens et al., 1982b; Rapaport et al., 1982; Pettigrew and Dreher, 1987; Tanaka et al., 1987; Guillemot et al., 1993; Tardif et al., 1997; Bergeron et al., 1998; Mimeault et al., 2002). However, in the present sample, inactivation did not have a clear effect on the presence and relative strength of suppressive regions of RFs.

#### Preferred Orientations

In our sample all neurons exhibited clear orientation selectivity. In the majority (73.5%; 11/15), PTV cooling did not induce clear changes in the optimal orientation. In particular, in all but four cells (4/15; 26.5%), the optimal orientations during inactivation were either the same or differed by no more than 15° from their control optimal orientations (Figures 3A,Bi,Bii). Note that only in one cell, the optimal orientation after rewarming PTV differed by more than 10° from that before inactivation of PTV (Figures 3Biii,iv).

#### Orientation-Tuning

Although in all area 19 cells, inactivation resulted in some changes in their orientation-tuning curves, substantial (≥20° change in WHH) widening (2/10 cells) or narrowing (1/10 cell) were rare (Figure 3Ci). Furthermore, in case of one of these cells, after rewarming of PTV there was still a substantial (>20°) difference in WHH of orientation-tuning with that in control run (Figure 3Cii). Overall, in cells in which inactivation caused widening of orientation-tuning curves, their mean WHH during inactivation was 55° (±34.2°) vs. 45.3° (±26.7°) before inactivation. In the case of cells in which inactivation resulted in the opposite effect, their mean WHH during cooling was 40.8° (±19.6°) vs. 47.4° (±18.7°) before inactivation.

#### Preferred Velocities

Consistent with numerous previous reports (Dreher et al., 1980; Duysens et al., 1982b; Rapaport et al., 1982; Dreher, 1986; Pettigrew and Dreher, 1987; see however Bergeron et al., 1998), in control condition, a large proportion (42%; 8/19) of area 19 cells preferred low (<15°/s) velocities and responded poorly to optimally oriented stimuli moving at velocities exceeding 20°/s (Figures 2A,B, 4Ci). Two of these cells with preferred velocities 6.6°/s and 13.2°/s, ceased to respond at velocities 46.2°/s and 66°/s, respectively. A majority (58%; 11/19) however, responded preferentially at velocities in the 40°–50°/s range (Figure 4Ci) and only one of them ceased to respond at the highest velocity (66°/s) tested.

In the majority (63%; 12/19) of cells, there was either no shift (48%; 9/19) or a very small downward shift (16%; 3/19) in preferred velocities during inactivation (Figure 4C). The RF centers of those cells were located within 10° from *areae centrales*.

However, in over a third of the sample (37%; 7/19), inactivation of PTV caused an upward shift in their preferred velocities (Figures 4A–C; control: 21.8 ± 19.1°/s; range: 1.1°–46.2°/s vs. during PTV inactivation: 35.4 ± 19.9°/s; range: 3.3°–52.8°/s). Furthermore, in two of these cells, their high cut-off velocities also shifted upward. The RF centers of all seven cells were located over 10° from *areae centrales*. It is worth noting that in all but one cell, after rewarming of PTV the preferred velocity was the same as that before cooling PTV (Figure 4Cii).

#### Direction Selectivity

Consistent with previous reports (Duysens et al., 1982a; Rapaport et al., 1982; Pettigrew and Dreher, 1987), most area 19 cells exhibited only a weak direction selectivity in control condition (low DSI—Figures 2A, 3Ai, 4A). Inactivation of PTV resulted in substantial changes in their DSIs (Figures 4A,B,Di). Thus, when DSI was calculated for velocities preferred before cooling (see **Preferred velocities**), a large proportion of cells exhibited substantial (≥20%) *increases* (21.5%; 6/28 cells) or *decreases* (6/28 cells) in their DSIs (Figure 4Di). Indeed, a couple of cells started with large (>70%) DSIs became absolutely direction selective (DSI = 100%) during inactivation (Figure 4Di). However, three cells that were weakly direction selective (DSIs < 40%) became completely non-direction selective (DSI = 0%) during inactivation (Figure 4Di). In addition, in a proportion (18%; 5/28) of cells, inactivation resulted in a 180° directional shift, that is, a complete reversal of their preferred direction (Figures 4A,Di**). Note that four of these cells also exhibited DSI changes of 20% or more. Overall however, low mean DSI, characteristic of area 19 neurons before cooling (mean: 48.81 ± 25.68%; range: 11.32%–100%, *n* = 28) was almost identical (*P* = 0.57, Wilcoxon test) with that (46.34 ± 34.09%; range: 0%–100%) during inactivation. Similarly, for the complex cells, the DSIs before PTV cooling (47.12 ± 26.69%, range: 11.32%–100%; *n* = 18) were not significantly (*P* = 0.62, Wilcoxon test) different from those (50.68 ± 27.96%, range: 0%–100%) during PTV cooling. However, for the simple cells, the DSIs in the control condition (49.44 ± 25.09%, range: 29.50%–100%; *n* = 9) were significantly (*P* = 0.04, Wilcoxon test) higher than PTV cooling (31.71 ± 40.22%, range: 0%–100%). Note that after rewarming of PTV, DSIs of most cells were much closer to these before PTV cooling (Figure 4Dii).

By contrast, when DSIs were calculated from the velocities optimal during cooling (see **Preferred velocities)**, most cells (8/10) exhibited greater DSIs. In most of them (6/8), the increases exceeded 20% (Figures 4A,B,Ei). Not surprisingly, the mean DSI during inactivation at 69% (±28.45; range: 18.2%–100%; *n* = 10) was substantially higher than that (44.25 ± 35.1%; range: 9.6%–100%) before inactivation. Note however, that after rewarming of PTV, the DSIs at stimulus velocities preferred during cooling remained quite similar to those during PTV cooling (Figure 4Eii).

#### Effect of Inactivation of PTV Cortex on Background Activities, Velocity Preferences and Direction Selectivities of Area 17 Cells

In a previous series of experiments (Huang et al., 2007), we have examined the effects of inactivation of PTV cortex on velocity-tuning of a small sample of area 17 neurons. However, the data were not included in our analysis of effects of inactivation of PTV cortex (Huang et al., 2007) and are included here for comparison.

The discharge regions of all area 17 neurons examined for the effect of inactivation of PTV cortex on velocity-tuning were located close to the horizontal meridian within 8° into the contralateral visual hemifield (Figure 1C).

Before inactivation, the mean background activity of simple cells at 0.23 spikes/s (±0.48; range: 0.01–1.33; *n* = 7) was almost identical to that (mean: 0.25 ± 0.50; range: 0.01–1.37) during inactivation. For complex cells, their control mean background activity (1.22 ± 1.98 spikes/s; range: 0.19–4.19; *n* = 4) was slightly higher than that (1.05 ± 1.55; range: 0.18–3.38) during cooling and substantially higher than that of simple cells (see Huang et al., 2007).

**Figure 5:**
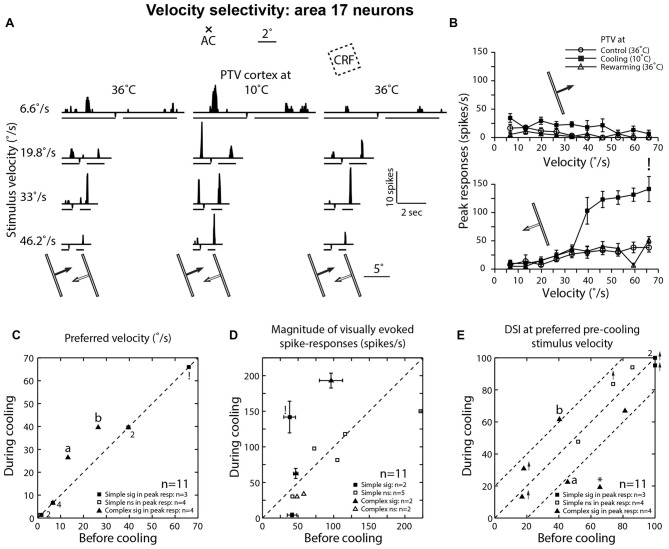
**(A)** Effects of PTV cooling on PSTHs of spike-responses of a class 2 area 17 complex cell to optimally oriented light bar moving at different velocities. Stimulus size: 10° × 0.4°. Note the bin widths for PSTHs for stimulus velocities 6.6°/s, 19.8°/s and 33°/s were 22 ms while those for the velocity 46.2°/s, the bin width was 6 ms. (**B)** Velocity-tuning of peak spike-responses of the same cell shown in **(A)**. Note that during PTV inactivation there is a substantial *increase* in the magnitude of responses as well as substantial increases in DSI especially at velocities of 6.6°/s and 19.8°/s. **(C)** Preferred velocities of area 17 cells before vs. those during cooling. Note also that in a couple of cells (a and b), PTV cooling resulted in upward shifts in the preferred velocities. Finally, note that the numbers indicate the number of data points that overlap. **(D)** Effects of PTV cooling on the magnitude of spike-responses of area 17 cells. ! In **(C)** and **(D)** denote the cell marked in lower panel of **(B)**. **(E)** DSIs of area 17 cells at stimulus velocities optimal before cooling. Note in three cells, cooling PTV resulted in large (≥20%) changes in their DSIs. *Indicates cell in which inactivation of PTV resulted in complete reversal of preferred direction.

In control condition, the mean peak-firing rate of area 17 neurons at 80.65 spikes/s (±54.45; range: 38.35–221.9; *n* = 11) was substantially higher than that of area 19 neurons in either control or PTV inactivation conditions (see above) but slightly lower than that (85.65 ± 60.05; range: 4.0–192.9) of area 17 neurons during inactivation of PTV (Figure 5D).

Before inactivation, a majority (63.5%; 7/11) of area 17 neurons preferred low (<15°/s) velocities (Figure 5C). During inactivation, an upward shift in preferred velocities was observed in two cells (both were complex; Figures 5A–C). Overall however, the mean preferred velocity of area 19 neurons during inactivation at 21.85°/s (±21.65; range: 1.4°–66°/s) was virtually the same as that (19.45 ± 20.95°/s; range: 1.1°–66°/s) before inactivation.

In one cell, PTV cooling resulted in a substantial increase in its firing rate during motion along the axis perpendicular to optimal orientation and this increase was accompanied by substantial stimulus velocity-linked changes in DSI (Figure 5E; see Figures 4A,B for area 19 neurons).

When DSIs were calculated for preferred velocities before cooling, in most cells there were substantial (≥20%) changes (Figure 5E). In one cell, inactivation resulted not only in a big reduction in DSI but also in a 180° directional shift (Figure 5E*). Overall however, the mean DSIs of area 17 cells before (72.15 ± 31.3%; range: 17.0%–100%; *n* = 11) and during (68.25 ± 34.5%; range: 13.2%–100%) inactivation of PTV cortex were quite high (see Duysens et al., 1982a).

## Discussion

### Cortical Regions Inactivated by Cooling Probe Confined to PTV Cortex

The temperature gradient in the cat visual cortex, measured along the center of the cooling probe, is consistently ~5°–6°C/mm (see for control experiments and review Huang et al., 2007; cf. also Casanova et al., 1997). Indeed, when the temperature of the probe confined to PTV was lowered to ~10°C, the tissue within 2 mm radius from the edge of the probe was between 10°C and 20°C. At this range, the generation of neuronal action potentials (spikes) becomes unreliable (Volgushev et al., 2000). Furthermore, in control experiments we (Huang et al., 2007) have found that when the probe was cooled to ~10°C, the spike-activity have largely ceased not only in PTV directly under the probe but also in parts of the cortex in the immediate proximity (2 mm) of the probe’s edge. Overall, the spike-activity silenced region included parts of areas 20a and 20b located on the ventro-lateral aspect of cerebral hemispheres, PS area (Updyke, 1986) and parts of areas 17 (V1), 18 (V2), 19 (V3) and 21b (Figure 1A). All areas 19 and 17 neurons in which the effects of PTV inactivation have been studied had their RFs located along the visual streak. In parts of areas 18, 19 and 21b (Figure 1A) affected by the spread of cooling, the upper contralateral visual field is represented (Tusa et al., 1979; Tusa and Palmer, 1980) while area PS contains representation of the lower contralateral visual field (Updyke, 1986). Feedforward and to a lesser extent, feedback connections between visuotopically organized cortical areas, appear to be confined to their visuotopically corresponding regions (Salin and Bullier, 1995; Dreher et al., 1996; Morley et al., 1997). Consistent with this, reversible inactivation of the higher-order areas does not seem to affect responses of neurons in topographically non-corresponding parts of the lower areas (Martinez-Conde et al., 1999; Huang et al., 2007). Nevertheless, in view of the fact that there are long-range intrinsic cortico-cortical connections in cat’s areas 17 and 18 (e.g., Gilbert, 1993; Dreher et al., 2001; Chavane et al., 2011), it is remotely possible that inactivation of parts of areas 18 and 19 in which the upper contralateral visual field is represented contributes somehow to the effects observed in the present samples of areas 19 and 17 neurons.

### Effects of Inactivation of PTV on the Magnitude of Responses of Area 19 Neurons

#### Excitatory and/or Suppressive Effects

Virtually all extrinsic afferents, including feedback cortico-cortical inputs to the cat’s primary visual cortex use glutamate and/or asparate as neurotransmitters and are thus presumably excitatory (Pérez-Cerdá et al., 1996). Indeed, electrical stimulation of areas 18 (V2) or 19 (V3) of the cat (Bullier et al., 1988) or the latero-medial (LM presumptive V2) area of the rat (Shao and Burkhalter, 1996) or area V2 of the mouse (De Pasquale and Sherman, 2011) result in the orthodromic, monosynaptic spike discharges or mono-synaptic depolarizations of neurons in their respective ipsilateral V1.

Consistent with the predominant excitatory nature of cortico-cortical feedback connections, reversible inactivation of area V2 (cat: Alonso et al., 1993b; Martinez-Conde et al., 1999; several species of monkeys: Sandell and Schiller, 1982; Bullier et al., 1996; Hupé et al., 2001a) results in reduction in spike-responses to optimized visual stimuli of a substantial proportion of V1 neurons.

In our study, inactivation of the entire ipsilateral PTV cortex resulted in the reduction (albeit rarely significant) of background spike-activities of simple and complex area 19 cells. Also during inactivation, in a quarter (significant) to half of the sample, the magnitude of spike-responses was reduced. Interestingly, inactivation of higher-order pattern/form-processing areas 21a and 19, reciprocally and strongly interconnected with area 17, results in reduction in the magnitude of spike-responses to optimal visual stimuli in a great majority of affected area 17 neurons (Wang et al., 2000). The excitatory nature of feedback cortico-cortical connections is not restricted to pattern/form processing stream. In particular, reversible inactivation of cat’s motion area—posterior middle suprasylvian cortex (Galuske et al., 2002) caused reduction in the signal strength in orientation and direction maps in the parastriate cortex (area 18, V2). Similarly, cooling motion-processing medio-temporal (MT) cortex of macaque monkeys results in reductions of magnitude of spike-responses of neurons in the ipsilateral areas V1, V2 and V3 (Hupé et al., 2001b).

The question arises if the visual signals arriving via feedback cortico-cortical connections can *per se* drive the spike-activity of cortical neurons. There is a clear evidence that in the cat, transient reversible inactivation of small volume of layer A of LGNd results in virtual silencing of simple and complex cells in area 17 whose RFs are in the precise retinotopic alignment with the center of GABA injections into the LGNd (Martinez and Alonso, 2001). On the other hand, when direct input from layer A of LGNd is reversibly blocked, the visuotopically corresponding part of cat’s area 18 is able to provide effective excitatory feedback drive to the supragranular layers of area 17 (Mignard and Malpeli, 1991). Overall, relatively selective removal of the feedback input from the higher-order cortical areas, without removing the other channels through which the visual input can reach a cortical neuron, might render the neuron unresponsive to visual stimuli. Thus, some of our area 19 neurons ceased to respond to visual stimuli when almost entire ipsilateral PTV cortex has been selectively inactivated, and their responsiveness to visual stimuli was restored when PTV was rewarmed. Similarly, when the entire ipsilateral area 18 (V2), rather than only the part corresponding visuotopically to the regions in V1 recorded from, was inactivated, some V1 neurons ceased to respond to visual stimuli (Sandell and Schiller, 1982). Their responsiveness to visual stimuli was restored when V2 was rewarmed (Sandell and Schiller, 1982).

Despite the excitatory nature of feedback projections: (1) reversible inactivation of circumscribed regions of superficial layers 2/3 of cat’s area 18 results in an *increase* in the magnitude of spike-responses of 33%–55% of neurons in layers 2/3 in the visuotopically corresponding part of area 17 (Martinez-Conde et al., 1999); and (2) in almost a third of the present area 19 sample, inactivation resulted in *increases* in the magnitude of spike-responses, and in ~10% of the sample those *increases* were significant. Most likely, the suppressive effects are conveyed by inhibitory local interneurons, intrinsic to the area recorded from. In rats, although 98% of feedback projections from area LM (presumptive V2) form excitatory synapses on excitatory neurons in area 17, ~2% make their connections with GABAergic interneurons (Johnson and Burkhalter, 1996). Although in the cat only a small proportion of the intracortical connections appear to be inhibitory (Gabbott and Somogyi, 1986; Shao and Burkhalter, 1996; Shevelev et al., 1998), almost every neuron in the cat primary visual cortex receives some inputs from local inhibitory interneurons (for review, see Eysel, 2002). In all mammals studied so far, 20%–30% of neocortical neurons are interneurons and most of them are inhibitory (DeFelipe, 1993; Markram et al., 2004). Significant increments in peak responses of area 19 neurons during inactivation are putatively due to the removal of excitatory input from PTV on GABAergic interneurons in area 19.

One has to be cautious in determining the overall proportions of area 19 cells receiving excitatory input vs. those receiving inhibitory input from PTV as they are not necessarily mutually exclusive. Thus, a number of area 19 cells appeared to receive an excitatory feedback input from PTV when their responses to optimally oriented stimuli were examined but appeared to receive suppressive feedback from PTV when the responses to sub-optimally oriented stimuli were examined (e.g., Figure 3Aii). Furthermore, the strength of suppressive feedback effects was substantially greater when the stimuli were moving faster (e.g., Figure 4; see also Figure 5 for area 17 cells).

Overall, it appears that feedback projections from PTV can exert excitatory and suppressive effects on spike-responses of area 19 neurons recorded from layers 3/4. It is possible that at least some of these effects are exerted via cortico-thalamic-thalamo-cortical loops (see Sherman, 2005; Sherman and Guillery, 2011). In the case of area 17 such loops would involve: PTV cortex—lateral posterior/pulvinar thalamic complex—area 17; in the case of area 19 it would involve: PTV cortex—lateral posterior/pulvinar thalamic complex—area 19 (Rosenquist, 1985; Dreher, 1986; Casanova et al., 1997; Piché et al., 2015). There is an important caveat to our conclusions due to the fact that we do not have information about the effects of inactivation of PTV on area 19 neurons in the superficial and deep layers.

### Effects of Inactivation of PTV on the RF Properties of Area 19 Neurons

#### Orientation Selectivity

Consistent with numerous earlier reports (Hubel and Wiesel, 1965; Duysens et al., 1982a, b; Rapaport et al., 1982; Dreher, 1986; Pettigrew and Dreher, 1987; Tanaka et al., 1987; Guillemot et al., 1993; Tardif et al., 1997), area 19 neurons exhibit clear orientation selectivity (see however Saito et al., 1988). Orientation selectivity of V1 neurons relies on multiple mechanisms, including mechanisms that are intrinsic to the area as well as the orientation biased inputs from the geniculate (for reviews, see Vidyasagar et al., 1996; Vidyasagar and Eysel, 2015) and feedback signals from layer 5 (Alonso et al., 1993b) or layers 2/3 (Martinez-Conde et al., 1999) of visuotopically corresponding parts of area 18 and/or higher-order visual cortices (e.g., Wang et al., 2000, 2010; Huang et al., 2007). It is likely, that in the case of area 19 neurons, orientation selectivity is to a large extent “inherited” via feedforward projections from the orientation selective neurons in the primary visual cortex. Nevertheless, in a small proportion of area 19 neurons, PTV inactivation caused clear (>10°) shifts in optimal orientation and/or substantial changes in the orientation-tuning widths. It is worth pointing out in this context, that in area 17 of anesthetized cats, shifts in preferred orientation not exceeding 15° do occur “spontaneously” (e.g., Henry et al., 1973).

#### Velocity Preferences and Cut-Off Velocities

It is commonly assumed that velocity preferences of visual neurons closely reflect the velocity preferences of geniculate neurons which provide their “driving” (Sherman and Guillery, 1998) excitatory input (e.g., Dreher et al., 1980; Orban et al., 1981; Duysens et al., 1982a; for review, see Orban, 1984). However, a substantial proportion of area 19 cells appear to receive Y type afferents from the LGNd (presumably responding to fast moving stimuli) do not respond to fast moving stimuli (Dreher et al., 1980). Similarly, some V1 neurons which receive geniculate afferents reliably activated by fast moving stimuli, presumably due to intracortical inhibition, do not generate action potentials when fast moving stimuli are presented (Goodwin and Henry, 1978). It is also worth noting in this context that: (1) the cut-off and preferred velocities of area 18 neurons tend to be much higher than those of most area 17 neurons (e.g., Dreher et al., 1980; Orban et al., 1981; for review, see Orban, 1984); and (2) reversible inactivation of circumscribed part of layer 5 of area 18 (V2), results in reversible upward shift in cut-off velocities and/or preferred velocities of a large (40%) proportion of layer 5 neurons in the visuotopically corresponding region of area 17 (Alonso et al., 1993a).

In this study, inactivation of PTV resulted in an upward shift in preferred velocities of some area 17 cells and in changes in their velocity preferences, with a tendency of a shift to higher velocities of over a third of area 19 neurons. Thus, it appears PTV cells providing the feedback projections to area 19 prefer higher velocities than the preferred velocities of area 19 cells based on the velocities preferred by the integrated input to area 19. Due to the fact that the highest velocity tested by us was 66°/s, and it was lower than the cut-off velocities of most area 19 neurons when PTV cortex was intact, we were unable to assess if inactivation of PTV resulted also in an upward shifts in the cut-off velocities of a substantial proportion of area 19 neurons. Nevertheless, in a couple of area 19 cells, cooling resulted in reversible upward shifts of their high cut-off velocities. Thus, the feedback from PTV might normally suppress the responses of area 19 cells to fast moving stimuli.

#### Direction Selectivity

The direction selectivities of cells in primary visual cortex of the cat are believed to be determined by a number of thalamo-cortical and intracortical mechanisms (for reviews, see Orban, 1984; Eysel, 2002; Humphrey and Saul, 2002). There is also substantial evidence indicating that in both carnivores and primates, feedback from the higher-order cortical areas affects the direction selectivity of neurons in the primary visual cortices. Thus, in the New-World squirrel monkey, during reversible inactivation of area 18 (V2), some direction selective cells in the ipsilateral area 17, lose most of their direction selectivity (Sandell and Schiller, 1982). In the cat, reversible inactivation of pattern/form processing area such as area 21a and a part of area 19 results in large (>20%) changes in DSIs of ~20% of neurons in the visuotopically corresponding part of area 17 (Wang et al., 2000). The increases in DSI of neurons in areas 19 and 17 (see also Huang et al., 2007) accompanying upward changes in velocity preferences during cooling of PTV, may be related to the fact that in the cat’s primary visual cortices the DSIs vary with velocities of stimulus motion (Orban et al., 1981; Orban, 1984; Humphrey and Saul, 2002).

The DSIs of neurons in the higher-order pattern processing areas might depend on some other factors. While normally neurons in area V4 (presumed homolog of area 21a of the cat) of behaving macaque monkeys exhibit very little direction selectivity, after prolonged exposure and adaptation to unidirectional motion of coherent random dot pattern, a third of the sample became strongly direction selective (Tolias et al., 2005).

#### Putative Role of Feedback Signals to Specific Cortical Areas

A large body of data indicates that dynamic changes in the magnitude of responses and RF properties of neurons in the lower cortical areas, including the primary visual cortex, are affected by the recurrent feedback signals from the higher-order cortical visual areas (see “**Discussion**” Section and review by Gilbert and Li, 2013). Hochstein and Ahissar (2002) proposed that high-acuity-yet analytical “vision with scrutiny” is based on the information derived from feedback-modified neuronal discharge activities of cells in the lower-order visual areas (“Reverse Hierarchy Theory”). Although there is a substantial degree of cross-talk between feedback pathways in both parallel information processing streams, the effects of inactivation of higher-order areas in the pattern/form processing stream vs. those in the motion processing stream are quite different. For example, in behaving cats, reversible inactivation of the ventral**-**posterior suprasylvian cortex, a region corresponding closely to PTV, results in severe impairment of retention of highly-familiar simple pattern discrimination when components of the patterns are stationary or in motion (Lomber et al., 1996a,b). By contrast, inactivation of motion processing areas lining the middle suprasylvian sulcus (for review, see Spear, 1991), affects discrimination of highly familiar simple patterns only when all components of the patterns are moving (Lomber et al., 1996b).

In anesthetized cat, silencing the feedback from motion processing areas in the middle suprasylvian region (for review, see Spear, 1991) tends to fairly selectively reduce the responsiveness of those area 18 neurons which exhibit a high degree of direction selectivity (Galuske et al., 2002). Furthermore, inactivation of feedback from motion processing middle suprasylvian region modulates direction selectivity but not orientation preferences of area 17 neurons (Shen et al., 2006). By contrast, inactivation of feedback from the pattern/form processing PTV, tends to shift upward preferred velocities and increase DSIs of areas 19 and 17 neurons (current study). Further the effects of inactivation of a particular higher-order area in a given processing stream on responses of neurons in a particular lower-order area in the same stream, are also quite specific. Thus, inactivation of area 21a affects strongly orientation (Wang et al., 2000; Tong et al., 2011) and spatial frequency tuning (Huang et al., 2004; Tong et al., 2011) but not direction selectivity tuning (Tong et al., 2011) of neurons in the ipsilateral area 17. It could be argued that feedback signals from the higher-order pattern processing areas, increase the “sparseness of coding” (Olshausen and Field, 2004) by neurons in the lower-order areas. Thus, feedback from area 21a enhances orientation selectivity of neurons in area 17 (Wang et al., 2007), while feedback from PTV suppresses responses of many area 19 and some area 17 neurons to faster moving stimuli (current study). This might reduce dynamic “cluttering” of visual scene during fast exploratory saccadic eye movements (Crommelinck and Roucoux, 1976; Blakemore and Donaghy, 1980) and allow the higher-order pattern processing areas to “concentrate” on processing visual information during small exploratory microsaccades, as well as slow drifts (Hebbard and Marg, 1960; Pritchard and Heron, 1960), plus slow disjunctive eye movements (Stryker and Blakemore, 1972) occurring during fixation of objects of interest. It appears that postulated (Pettigrew and Dreher, 1987; Guillemot et al., 1993) strong involvement of area 19 in stereoscopic analysis of the visual scene is restricted to analysis of static, rather than moving, interocular positional disparities (Mimeault et al., 2002).

It appears that irrespective of the information processing stream in which any given areas are “embedded”, feedback from a particular higher-order cortical area modulates those properties of neurons in the lower area, which play important roles in the information processing in the higher-order areas. Thus, cortical areas in the middle suprasylvian region want to “hear” about the direction of stimulus movement and their feedback signals enhance the magnitude of responses of strongly direction selective cells in the lower areas. By contrast, the pattern/form processing PTV cortex wants to “hear” about the specific properties of stationary/slowly moving stimuli and by feedback signals suppresses the responses of cells in the lower areas such as area 19 or area 17 to fast moving stimuli.

## Conclusion

In about a third of the sample of area 19 neurons, inactivation of higher-order pattern-processing PTV cortex resulted in significant changes in the magnitude of their peak responses to optimally oriented elongated moving bars. Only occasionally, silencing feedback signals from PTV resulted in an abolishment of the spike-responses of area 19 neurons. In a small proportion of area 19 cells, feedback from PTV appears to play a modulatory role in relation to such RF properties as optimal orientation and/or width of orientation-tuning curves. In a large proportion of area 19 cells and a proportion of area 17 cells, feedback from PTV plays a modulatory role in relation to stimulus velocity preferences and/or direction selectivity, that is, the properties which are usually believed to be determined by the inputs from the dorsal thalamus and/or feedforward inputs from the primary visual cortices. Thus, apparent specialization of area 19 for processing information about stationary/slowly moving visual stimuli is at least partially determined, by the feedback from the higher-order pattern-processing visual area.

## Author Contributions

JYH, CW and BD conceived, designed and conducted experiments and collected the data; JYH analyzed all of the data with assistance from CW, BD; JYH and BD wrote the article; all authors worked on the final draft of the article.

## Funding

This work was supported by a grant from the National Health and Medical Research Council of Australia.

## Conflict of Interest Statement

The authors declare that the research was conducted in the absence of any commercial or financial relationships that could be construed as a potential conflict of interest.

## Abbreviations

CRF: classical receptive field
DSI: direction selectivity index
LGNd: dorsal lateral geniculate nucleus
PSTH: peri-stimulus time histogram
PTV cortex: postero-temporal visual cortex
RF: receptive field
WHH: width at half-height.
